# Morphological diversity and fruit production of wild *Salacia kraussii* (*Celastraceae*) on the Northern Coast of KwaZulu-Natal, South Africa

**DOI:** 10.1016/j.heliyon.2024.e25332

**Published:** 2024-02-15

**Authors:** Merveille Mukaya Kisepa, Elijah Godfrey Zharare, Clemence Zimudzi, Arindo Lukawu Akweni

**Affiliations:** a Department of Agriculture, Faculty of Sciences, Agriculture and Engineering, University of Zululand, South Africa; b Faculté des Sciences Agronomiques, Université de Kikwit, Democratic Republic of the Congo; c Faculty of Biology, University of Zimbabwe, Zimbabwe

**Keywords:** Fruit production, KwaZulu-Natal, Morphological diversity, Morphotype, *Salacia kraussii*

## Abstract

This study aimed to assess morphological diversity within *Salacia kraussii*, a fruit and medicinal wild plant species, based on morphological features and compared the fruit production among morphological types (morphotype) that naturally occur on the northern coast of KwaZulu-Natal. Following one species plant survey, a description of the qualitative morphological features revealed that *S. kraussii* individuals mainly differed in their leaf shapes, having elliptic, oblong, or obovate leaves. That led us to the identification of three morphotypes, namely *Salacia kraussii ‘elliptic’, Salacia kraussii ‘oblong’* and *Salacia kraussii ‘obovate’*. The analysis of variance (one-way ANOVA) of plant quantitative features indicated that plant height, stem diameter, branch number, leaf number and area, and fruit number were significantly different between plants from different sites (p-values < 0.05) and morphotypes (p-values < 0.01). Generally, *S*. *kraussii* grows in KwaZulu-Natal as a suffrutex with many stems and exhibits short plant height, small stem diameter, branches, and little foliage per stem. The average fruit number recorded per plant stem was likewise few. Plants growing in Sikhalasenkosi (site1) dominated in average plant height (35.58 cm), leaf number (45), number of branches (4), and number of fruits (5). Plants with elliptic leaves constantly dominated in average plant height (34.45 cm), foliage (36 leaves of 16.29 cm^2^ each), number of branches (4), and number of fruits (5). A few plants exhibited a strong vegetative vigor and produced more than 20 fruits. There was a highly positive correlation (CC = 0.8) between plant height and leaf number, branch number and leaf number, and branch number and fruit number. However, a negative correlation (CC = −0.1) was recorded between the leaf area and stem diameter. Overall, the study showed wide morphological diversity and fruit production within and between populations of S. *kraussii*, on the northern coast of KwaZulu-Natal.

## 1 Introduction

Wild edible fruits play a significant role in meeting the nutrition and food security of many rural communities worldwide [[1], [2], [3], [4]]. Many people around the world continue to experience food insecurity as well as hunger (https://www.fao.org/faostat/fr/#data/FS). A healthy and balanced diet is an important part of maintaining good health and implies the consumption of meals that are high or rich in fruits and vegetables [[5], [6], [7]]. Access to commercialized fruits is a daily challenge in developing regions particularly in Africa and Asia. In these regions more than 70 % of rural families are dependent on wild fruits [8] for energy, vitamins, and dietary fiber intakes. In this case the wild fruits help to provide food diversity, balanced diet and to prevent malnutrition and other diet related disease [[9], [10], [11], [12], [13]].

Promoting the cultivation of wild edible fruits species as commercial crops is central to establishing food security, empowering local market actors, diminishing poverty in the rural areas and reducing fruit importation at national level [14,15]. Many studies highlighting the nutritional values of some known wild fruit species has been published worldwide. However, the genetic diversity of most of these species and their production traits that can be considered for developing commercial varieties for cultivation are not documented [16]. This is a major limitation to selection of useful species for incorporation in the food production system [8].

*Salacia kraussii,* is a wild shrub growing primely in the tropical and subtropical biomes, distributed from South Mozambique, Zimbabwe to KwaZulu-Natal in South Africa (https://powo.science.kew.org/taxon/urn:lsid:ipni.org:names:162612-1). The potential of using the fruit in jam and jelly manufacture has been reported [17]. The fruits, contain a low to moderate percentage of reduced sugars, with glucose averaging 3.7% and fructose averaging 3.9% [18]. This makes the fruits suitable for consumption by people with hyperglycemia, diabetes, and obese conditions. Its leaves possess antibacterial and antioxidant properties [19], while its roots have antimalarial properties, as well as being cytotoxic to HT-29 cell lines [20]. This reveals that *S. kraussii* contributes to food security and wellbeing of population of rural household that use its fruits and presents the potential for commercialization, reducing poverty in rural households and use as medicine in various diseases.

Currently, there is no data available on the morphological diversity and fruit production of *S.kraussii*, which can guide the selection and genetic improvement of the plant for use as a commercial horticultural crop. A wild plant taxon may present various morphological features that can confer to it multiple uses as horticultural crop. It is therefore worthwhile exploring first its morphological diversity and evaluate Its fruit production in the wild conditions. Important traits considered in the selection of wild edible plants for agricultural production are firstly morphological, such as the number, size, and weight of edible parts like fruits, and secondly, their value as medicines or other industrial uses [[21], [22], [23], [24], [25], [26], [27], [28]]. Some of these features vary considerably within plant species and can be separated into different morphotypes. A plant morphotype is referred to as a group of plants of a species that is distinguished from other groups of the same species by a particular set of characteristics; nonetheless, these groupings do not reflect a formally recognized taxonomic rank [29]. A taxon having numerous morphotypes is considered rich in diversity, which can confer upon it multiple uses based on the traits of each morphotype [30]. Morphological characterization is a traditional and efficient method for rapidly determining the diversity expressed through variation of phenotypic features within a particular taxon [[31], [32], [33], [34]]. This assists plant breeders and producers in their selection of wild species morphotypes with desired traits to maximize their profit and improve and develop high-performing varieties [35]. For the optimal utilization of wild plants, comprehensive identification of the morphotypes in the relevant taxa is required.

This study aimed to document the morphological diversity in wild *S. kraussii* plants growing on the northern coast of KwaZulu-Natal (KZN) and determine the fruit production of each morphotype identified. The study particularly focused on the variations of qualitative and quantitative morphological features and fruit production among *S. kraussii* plants in temperate and semi-arid sites.

## 2 Materials and method

### 2.1 Study area

**Fig. 1:**
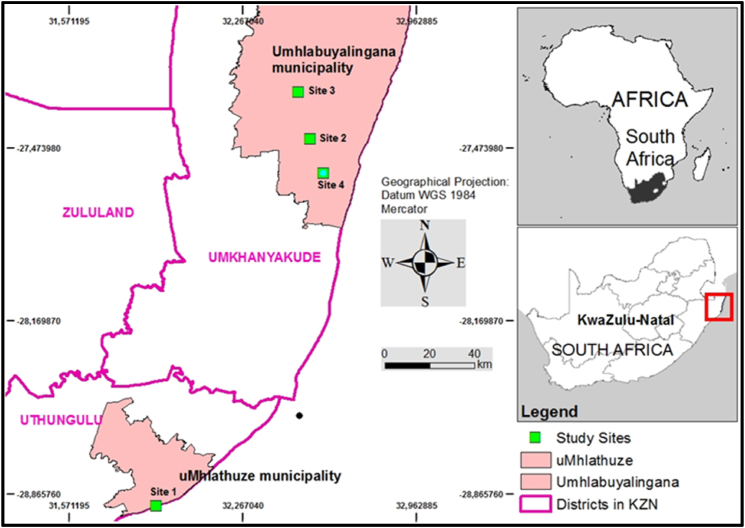
**Study area**: Spatial location of the research sites including Sikhalasenkosi (Site1), KwaNdongeni (Site2), Enhlambeni (Site3) and KwaMbila (Site4) in the Province of KwaZulu-Natal.

This study was conducted as a series of plant surveys on the northern coast of KZN, in South Africa. Generally, in KZN, the maximum temperature in the hottest month exceeds 35 °C whilst the minimum temperature in the coldest month is below 14 °C. The area is dominated by sandy soils and wooded savanna [36,37]. The study area is divided into six macroclimates. However the surveys were conducted at four sites in only two of the microclimates namely: (i) the arid–hot steppe (*BSh*), known as semi-arid zone, and (ii) the temperate zone described as having a hot summer and -without a pronounced dry season (*Cfa*) [[38], [39], [40], [41]]. The four sites included Sikhalasenkosi (Site1), KwaNdongeni (Site2), Enhlambeni (Site3) and KwaMbila (Site4) (Fig. 1). Site1 is in King Cetshwayo district (formerly uThungulu district), located in the temperate zone (*Cfa*) however, sites 2,3 and 4 are in uMkhanyakude district, in the semi-arid zone (*BSh*) (Table 1) [38,39]. In terms of land use, the study sites are used as grazing areas by surrounding communities who occasionally use fire to control woody species and to stimulate grass growth.

**Table 1:** **Geographic coordinates and climate characteristics**: ***Cfa*** (Temperate hot summer without dry season)**, *BSh*** (Arid hot steppe or Semi-Arid), **T (**Temperature in ^**o**^**C**)**)**.

DISTRICT	Locality	Site	Geo. Coordinates	C.Z^a^	T^a^	P
King Cetshwayo	Sikhalasenkosi	1	28°54′47.5″S 31°55′01.3″E	*Cfa*	b>22	1200^b^
uMkhanyakude	KwaNdongeni	2	27°26′17.6″S 32°32′12.0″E	*BSh*	a>18	500^c^
uMkhanyakude	Enhlambeni	3	27°14′57.7″S 32°29′21.8″E	*BSh*	a>18	500^c^
uMkhanyakude	KwaMbila	4	27°34′29.7″S 32°35′23.6″E	*BSh*	a>18	500^c^

a (Mean annual temperature**)**.

b (Temperature in hottest month), **P** (High annual precipitation in mm). a[38],c [42].

c (https://www.umhlathuze.gov.za/index.php?option=com_content&view=article&id=73&Itemid=338)

### 2.2 Soil analysis

Soil samples were collected in each study site at 25 cm depth soil and 200g of each collected sample were separately packed in cartoon containers. Soil analysis was performed at the Soil Fertility and Analytical Services Section of the KwaZulu-Natal Department of Agriculture and Rural Development as followed: Total Carbon, Nitrogen and Sulphur were analyzed by the Automated Dumas dry combustion method using a LECOCNS 2000 and the Organic carbon by the Walkley-Black method. This method involved weighing soil samples into a ceramic crucible to which 0.5g of vanadium pentoxide was added. The crucible was introduced into a horizontal furnace, where the sample was burned in a stream of oxygen at 1350 °C. The gases produced were passed through two infra-red cells where the Sulphur (as SO2) and carbon (as CO2) were determined. Nitrogen is determined (as N2) in a thermal conductivity cell. Organic carbon by the Walkley-Black method where the organic matter is oxidized by potassium dichromate in a sulphuric acid medium. The excess dichromate is determined by titration with standard ferrous sulphate solution. The particle size of soils and soil texture, suspended clay and fine silt were determined by dispersion sedimentation technique which consisted of to treat 20g soil sample with hydrogen peroxide to oxidize the organic matter. The sample was made up to 400 ml with de-ionized water and left overnight. The clear supernatant was siphoned off and the sample puddled. A further addition of de-ionized water was added, the sample stirred and left overnight. The clear supernatant was again siphoned off. Dispersing agents (NaOH and sodium hexametaphosphate) were added, and the sample stirred on Hamilton Beach stirrers. The suspension was made up to 1 L in a measuring cylinder and the clay (<0.002 mm) and fine silt (0.002–0.02 mm) fractions measured with a pipette after sedimentation and Sand fraction was estimated by difference. Once the particle size distributions of the two soils are known, their textural class is determined from a diagram (Textural triangle) defining particle size limits of the various textural classes.

### 2.3 Plant morphological description

At each site, survey data on the wild *Salacia kraussii* were collected in a plot (quadrat) of 250 m^2^ (25 m × 10 m). Plots were delimited with a tape measure (model Fibreglass Open Frame 50 m) in areas with a high plant density of *S. kraussii,* and the number of plants within each plot was recorded. Qualitative features such as plant habit, stem and branching pattern, leaf type, arrangement, shape, lamina color and texture, leaf margin, apex and base shapes, inflorescence type, flower color, ovary insertion, petal, sepal, carpel and stamen traits of each plant within the plot were described and determined by matching the plant parts features with models represented in the leaf architecture manual and the plant's glossaries simultaneously [[43], [44], [45], [46]]. There were three morphotypes identified based on qualitative data. Quantitative features such as plant height, stem diameter, branch number, leaf number, length and width and fruit number were measured on a random sample of 300 plants of which 25 plants were for each of the three morphotypes at the four sites. Plant heights were determined with the tape measure while leaf length and width were measured using a metric ruler (model Stainless Steel 300 mm) and the stem diameter, with a caliper (model Kendo, 150 mm; 0.05 mm precision). The approximate leaf area was determined using the following formula**:** S=0.75(Ll**).** Where $\prime \prime S^{\prime \prime }$ denoted the leaf surface, $\prime \prime L^{\prime \prime }$ is the whole length of the leaf lamina from the apex to the base extremity, and ″l", is the width at the medium of the lamina [43]The number of fruits per plant was established by counting them. All morphological data collected for each site were entered in a field sheet giving the date, time of collection, and geographic coordinates of the site (via Google Maps); and were recorded in Excel files for statistical analysis.

### 2.4 Statistical analysis

Analysis of variance (ANOVA) was conducted using R-software (R-version 4.2.2) to determine the differences between means of quantitative data. One-way ANOVA was used to express the statistical significance between different plant quantitative features, based on a single study variable analysis such as the site or morphotype. A two-way ANOVA was used to analyze each quantitative feature variation based on combined morphotype and study site variables. A correlation analysis was conducted to describe the relationship between the plant's quantitative features.

## 3 Results

### 3.1 Soil characteristics

The analysis of soil samples collected in the survey areas showed that all sites were characterized by sandy soil, with acidic pH (4.47–5.98) low in nitrogen and organic carbon (Table 2). The amount of nutrients at site 1 was higher than other sites, with 0.11 mg/L, 27 mg/L, 78 mg/L and 1.0% of nitrogen, phosphorus, potassium, and organic carbon respectively. Sites 2, 3 and 4 had 0.05–0.07 mg/L of nitrogen, 5–7 mg/L of phosphorus, 20–56 mg/L of potassium and less than 0.05 % of organic carbon (Table 2).

**Table 2:** **Soil characteristics**: O.C(Organic Carbon), N(Nitrogen), P(Phosphorus) and K(Potassium).

Locality	Site	Soil Texture	Clay (%)	O.C (%)	N (%)	P (mg/L)	K (mg/L)	pH (KCl)
**Sikhalasenkosi**	1	Sandy	<5	1.0	0.11	27	78	5.98
**KwaNdongeni**	2	Sandy	<5	<0.5	<0.05	7	56	4.74
**Enhlambeni**	3	Sandy	<5	<0.5	<0.05	7	20	4.47
**KwaMbila**	4	Sandy	6	<0.5	0.07	5	41	4.8

### 3.2 Plant qualitative features

**Fig. 2:**
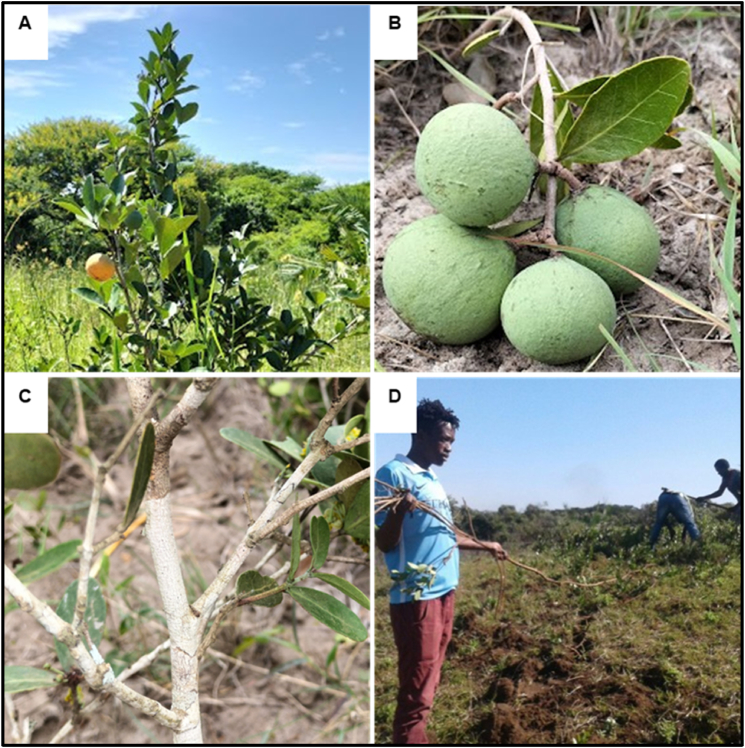
**Plant habit**: Erect upright stem of about 1.5 m (A). A short upright creeping stem bearing fruits (B). An upright stem with many branches (C). A rhizome of about 10 m underground (D).

*Salacia kraussii* grows naturally as a suffrutex on sandy soils in savannah vegetation along the coast of the Indian Ocean on the northern coast of KZN. The plant is characterized by either an erect (Fig. 2A) or creeping stem (Fig. 2B). Due to their short height, *S. kraussii* plants are barely noticeable from a distance in the grass. Nevertheless, during fruiting, the presence of fruit increases their visibility. Generally, plants produce a rhizome system, from which are developed multiple upright stems which subsequently produce one to many branches (Fig. 2C). The rhizome may exceed 1 m underground (Fig. 2D). The height of the highest upright stem of each plant is considered here as the total height of the referred plant.

**Fig. 3:**
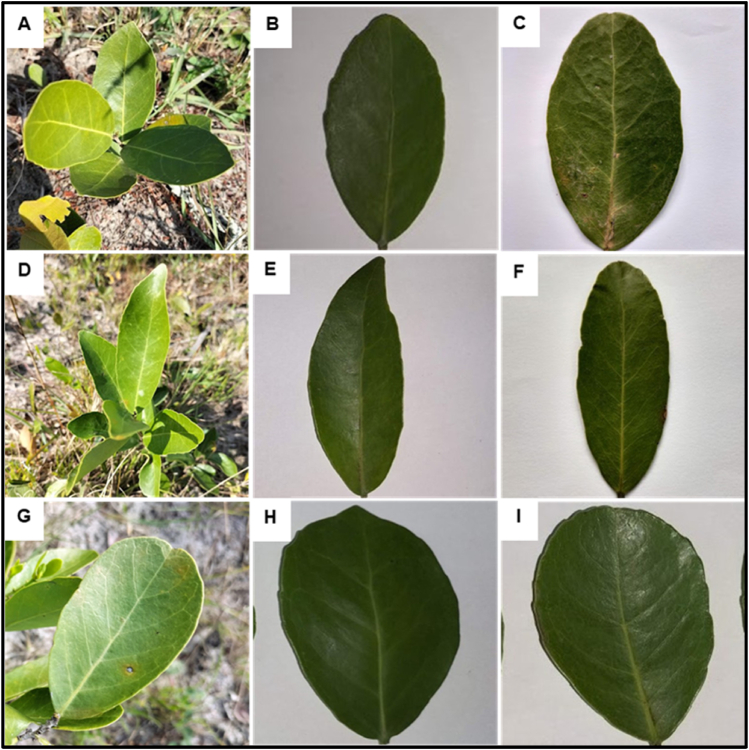
**Variation in leaf shape among *S. kraussii* plants**: sample with elliptic leaves (A), with obtuse (B) or a slightly rounded apex (C). Sample with oblong leaves (D), with an acute (E) or a rounded apex (F). Sample with obovate leaves (G), with a mucronate (H) or a rounded apex (I).

*S*. *kraussii* plants have simple petiolate (semi-terete) leaves, alternately to sub-oppositely arranged on the stem. Three leaf shapes were observed i.e., elliptic, oblong, and obovate leaves (Fig. 3). For all leaf shapes, laminas were green, coriaceous, shiny above but pale to dull below, entire, or serrate (slightly teethed). The elliptic leaf shape (Fig. 3A) possesses an obtuse (Fig. 3B) or a slightly rounded (Fig. 3C) apex; both patterns were observable on one single plant. The oblong leaf (Fig. 3D) possessed an acute (Fig. 3E) or a rounded apex (Fig. 3F) while the obovate leaf (Fig. 3G) possessed a mucronate (Fig. 3H) or rounded apex (Fig. 3I). The above leaf shapes were found on separate plants, however, the apex shape relative to each leaf shape varied among leaves of the same plant. The leaf base shape was convex on all the leaf shapes.

**Fig. 4:**
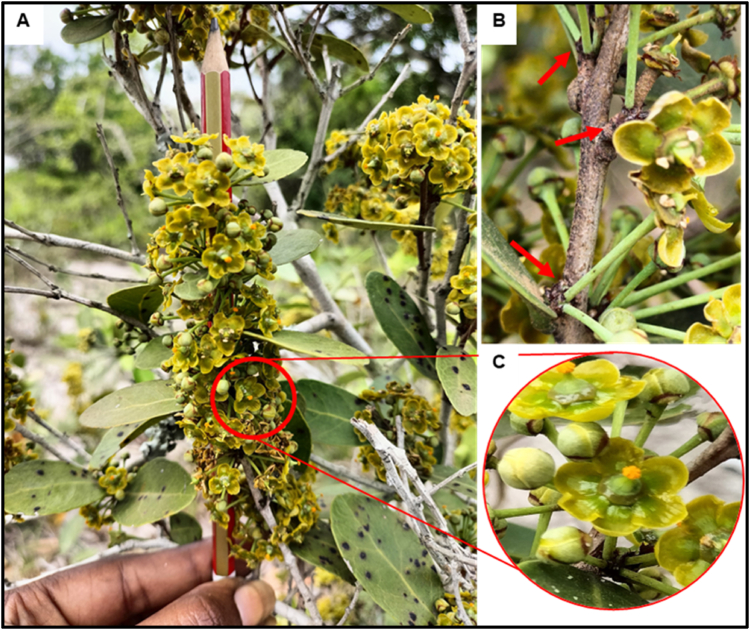
***S. kraussii* inflorescence**: An axillary fascicle of many yellow to greenish-yellow flowers (A). Flowers are inserted on the stem at the leaf buds. A single flower with 6 petals (C).

The inflorescence is an axillary fascicle bearing yellow to greenish-yellow bisexual flowers (Fig. 4A). Flowers are inserted at the inner face of the leaf buds (Fig. 4B). Flowers bear 5 to 7 petals, 3 to 4 sepals, a super ovary surrounded by a conic-cylindric disk and 3 stamens with orange pollen (Fig. 4C). Flowering time may last up to 10 weeks starting by the end of June or July. The inflorescence characteristics were the same in plants with different leaf shapes.

**Fig. 5:**
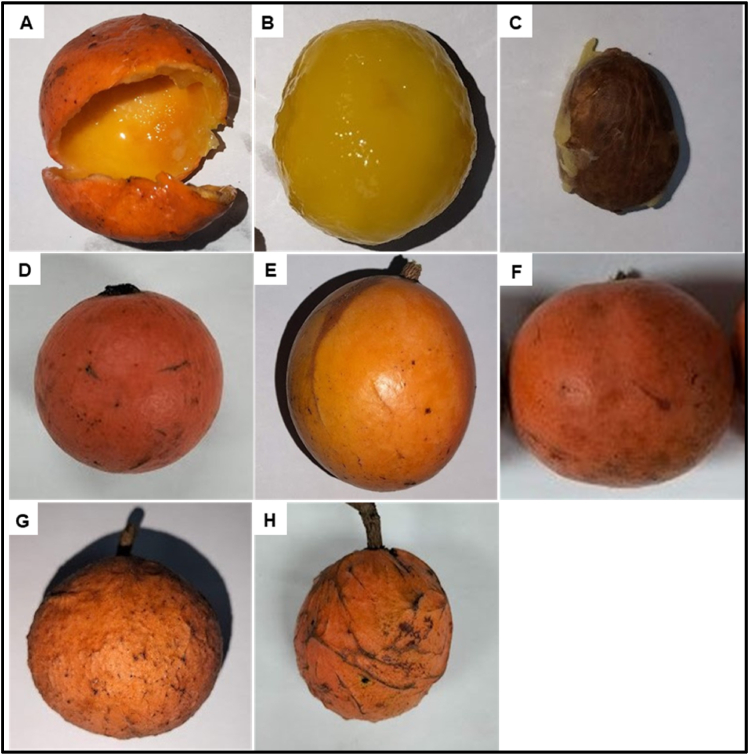
***S. kraussii* Fruit Components and morphology**: Peel (A), pulp (B) and seed (C); ellipsoid (D), ovoid (E) and slightly bilobed (F) fruit shapes; Soft (D, E, F), rough (G) and striped (H) fruit epicarp textures.

The fruit is a green berry which ripens into an orange color. The fruit peel is a thick epicarp (Fig. 5A), which envelops one or many yellow and gelatinous mesocarps, which constitute the edible pulp (Fig. 5B). The pulp surrounds a single seed (Fig. 5C). There can be up to 5 seeds within a single fruit. The fruit may have an ellipsoid (Fig. 5D), ovoid (Fig. 5E), or a slightly bilobed shape (Figure F). The epicarp texture varies from one fruit to another, being soft (Fig. 5DE, and F), rough (Fig. 5G and H), or striped (only one pattern was seen) (Fig. 5I). There was no consistent difference in fruit morphology among the morphotypes.

The leaf shape appeared to be the main morphological difference among *S. kraussii* plants and this necessitated identification of three morphotypes *i.e*: *Salacia kraussii ‘elliptic’* (Elliptic), *Salacia kraussii ‘oblong’* (Oblong) and *Salacia kraussii ‘obovate’ (Obovate*). The leaf shape was therefore considered the foremost qualitative criterion to study variation in plant quantitative features among S. *kraussii* populations located in different sites.

### 3.3 Abundance of each morphotype per site of study

Observations based on plots’ plants in the four sites showed that the morphotype Obovate was the most dominant with a total of 152 (36.45%) plants, followed by Elliptic with 139 (33.33%) plants and Oblong was the least with 126 (30.22%) out of 417 total plants studied. Site 3 plot had the highest plant density with 113 (27.10%) and site 4 had the least with 96 (23.2%) out of 417. The Obovate morphotype at site 3 represented the biggest proportion of plants of all morphotypes and all study plots with 53 plants while the Oblong morphotype at site 4 had the least proportion with 25 plants per plot (Table 3).

**Table 3:** **Plant Overview in the for sites:** Total population, number (Nbr) and percentage (%) of plants of each morphotype of *S. kraussii* per plot (250 m^2)^), in the four sites.

MORPHOTYPES	Elliptic		Oblong		Obovate		TOTAL/Site	
	Nbr	(%)	Nbr	(%)	Nbr	(%)	Nbr	(%)
Site 1	39	35.78	23	21.10	47	43.1	109	26.14
Site 2	26	26.26	48	48.48	25	25.25	99	*23.74*
Site 3	34	30.09	26	23.01	53	*46.90*	113	27.10
Site 4	40	41.67	29	30.21	27	*28.13*	96	23.02
TOTAL/Morphotype	**139**	**33.33**	**126**	**30.22**	**152**	***36.45***	**417**	**100**

### 3.4 Dispersion analysis of plant quantitative features based on single variable (one way-ANOVA)

#### 3.4.1 Variation of plant quantitative features among the four sites

**Fig. 6:**
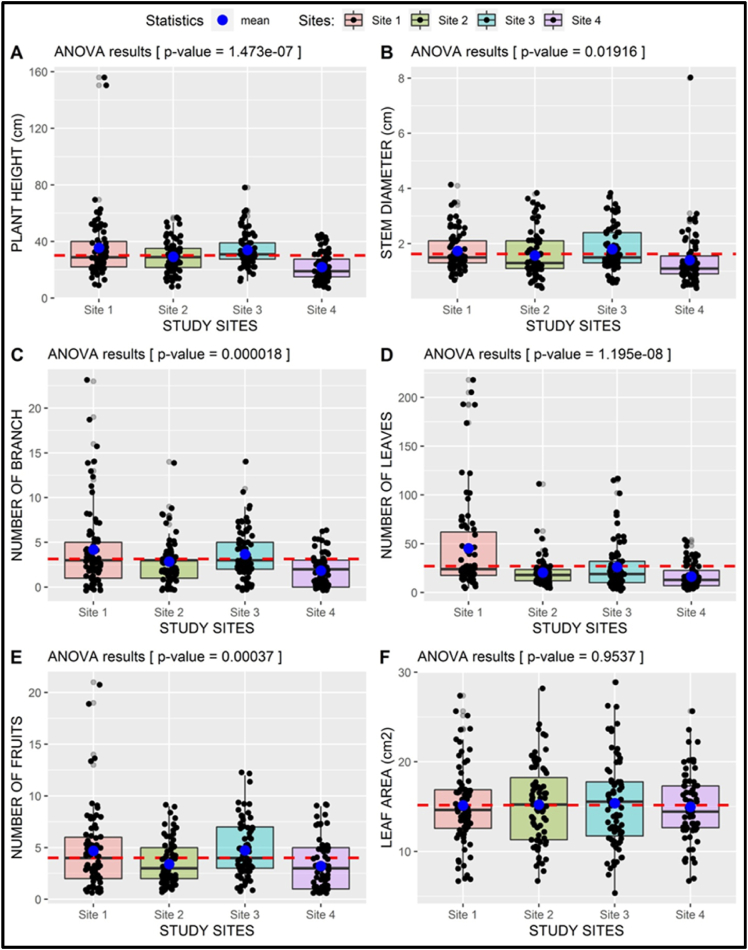
**One-way ANOVA of *S. kraussii* quantitative features by site**: Plant height (A), stem diameter (B), branch number (C), leaf number (D), fruit number (E) and leaf area (F). The p-value summarizes the results of the ANOVA test. The red-dotted line indicates the overall mean value of the involved plant parameter and the horizontal black lines inside the boxes are the median values.

The results (Fig. 6) demonstrated that quantitative features namely the plant height, stem diameter, branch number, leaf number, leaf area and fruit number varied between study sites. The plant height at site 1 was the tallest, with an average height of 35.58 cm. They were followed by those at sites 3, 2, and 4 with mean heights of 33.97 cm, 29.20 cm, and 21 cm, respectively (Fig. 6A). Site 3 had the largest stem diameter, measuring 1.80 cm, while those at site 4 had the smallest diameter, measuring 1.40 cm on average per plant. Site 1 and 2 stems measured 1.74 cm and 1.57 cm in diameter respectively (Fig. 6B). Site1 was dominated by plants with 4 (4.20) branches per stem, the number of plants with 4 branches per stem decreased among the sites in the order site 3, site 2, and site 4, the respective number of branches being 4 (3.68), 3 (2.85), and 1 (1.32) per stem (Fig. 6C). Similarly, site 1 plants had the most leaves followed by those at sites 3, 2, and 4, with an average of 45 (45.23), 26 (26.15), 21 (20.52), and 16 (16.35) leaves per plant respectively (Fig. 6D). Site 3 plants had the greatest average leaf area which amounted to 15.37 cm^2^ average, followed by site 2 with 15.19 cm^2^, site 1 with 15.13 cm^2^, and site 4 with 14.96 cm^2^ (Fig. 6F). The least average fruit produced per plant was 2, which was recorded at site 4. The number of fruits per stem increased at sites 2,3 and 1 to 3, 4, and 5 fruits per plant, respectively (Fig. 6E). The 4 sites differed significantly in total plant height (p-value = 1.473 ˣ 10^−7^), stem diameter (p-value = 0.01916), branch number (p-value = 1.8 ˣ 10^−5^), number of leaves (p-value = 1.195 ˣ 10^−8^) and number of fruits (p-value = 3.7 ˣ 10^−4^) but did not differ significantly in leaf area (p-value = 0.9537) (Fig. 6).

#### 3.4.2 Variation of plant quantitative features among *S. kraussii* morphotypes

**Fig. 7:**
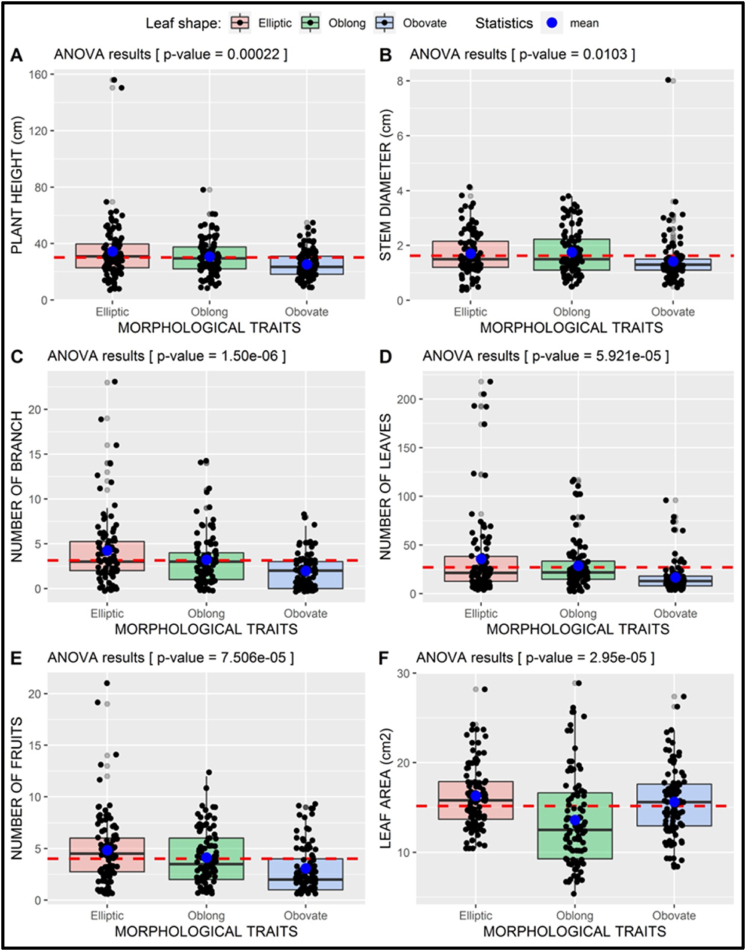
**One-way ANOVA of *S. kraussii* quantitative features by morphotype**: Plant height (A), stem diameter (B), branch number (C), leaf number (D), fruit number (E), and the leaf area (F). The red-dotted line indicates the overall mean value of the involved plant parameter and the horizontal black lines inside the boxes are the median values.

Similarly, to sites-based analysis, the result from the ANOVA (Fig. 7) demonstrated that quantitative characteristics varied among the *S. kraussii* morphotypes. The morphotypes differed significantly in the plant height (p-value = 2.2 ˣ 10^−4^), the stem diameter (p-value = 0.0103), the number of branches (p-value = 1.50 ˣ 10^−5^), the number of leaves (p-value = 5.921 ˣ 10-5), leaf area (p-value = 2.950 ˣ 10-5) and the number of fruits (p-value = 7.506 ˣ 10^−5^). The Elliptic morphotype had an average plant height of 34.45 cm, while those of the Oblong and Obovate morphotypes were 30.73 cm and 25.28 cm, respectively. The highest plant height of 156 cm was recorded in the Elliptic morphotype (Fig. 7A). The Oblong morphotype had the largest average stem diameter of 1.75 cm, followed by the Elliptic and Obovate morphotypes measuring 1.70 cm and 1.42 cm, respectively. Despite having a lower average than the others, the Obovate morphotype had generally the largest stem diameter measuring 8 cm (Fig. 7B). The maximum number of branches recorded per stem in the Elliptic morphotype was 4 compared to 3 and 2 branches for the Oblong and Obovate morphotypes respectively (Fig. 7C). The Elliptic morphotype had the most abundant foliage as evidenced by the greatest leaf number (36 leaves per stem) and the largest leaf size (16,29 cm^2^ per stem). In second place was the Oblong morphotype with 29 leaves per stem which provided a leaf area of 15,63 cm^2^ per stem. The Obovate morphotype, which came last, had 17 leaves and an average area of 13.60 cm^2^ per stem, which is less than the average of 27 leaves and a leaf area of 15.17 cm2 for all morphotypes (Fig. 7D and F). The Elliptic morphotype was the most productive in terms of fruit number. It had an average fruit count of 5 per plant. In contrast, the Oblong and Obovate morphotypes produced on average 4 and 3 fruits, respectively. The maximum number of fruits obtained in a plant of the Elliptic morphotype was 21, while those of the Oblong and Obovate morphotypes were 12 and 9, respectively (Fig. 7E).

### 3.5 Dispersion analysis of quantitative features based on combined two variables (two-way ANOVA)

**Fig. 8:**
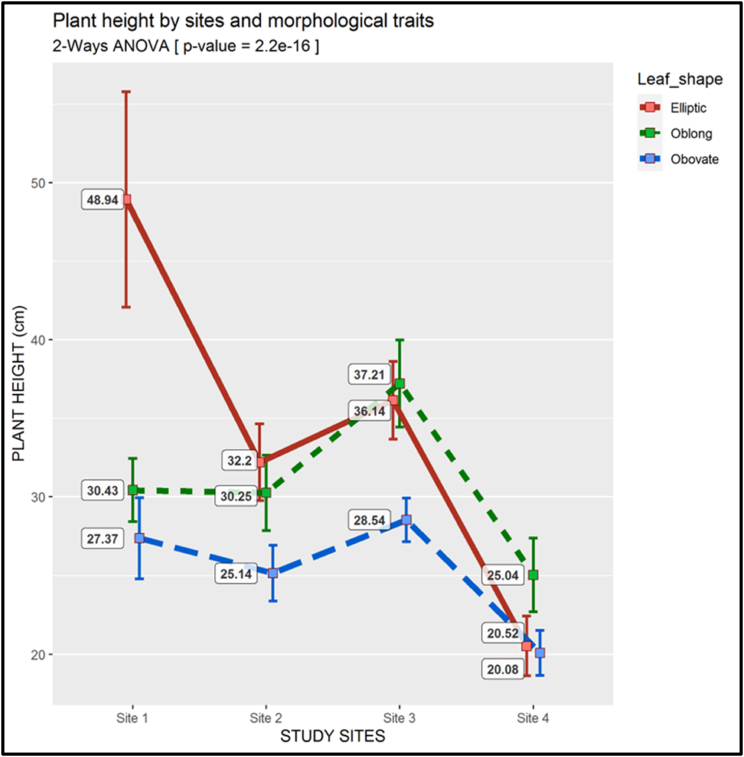
Two-way ANOVA of the Plant Height (There is a descriptive legend on the figure)

The combined analysis of plant height per morphotype and site of study showed that the Elliptic morphotype had the highest plant height at site 1 (mean = 48.98 cm) and site 2 (32.2 cm), but the Oblong morphotype had the highest mean height at site 3 (37.21 cm) and site 4 (25.04 cm); the Obovate morphotype had significantly the lowest stem height average at all the four sites (p-value <0.01) (Fig. 8).

**Fig. 9:**
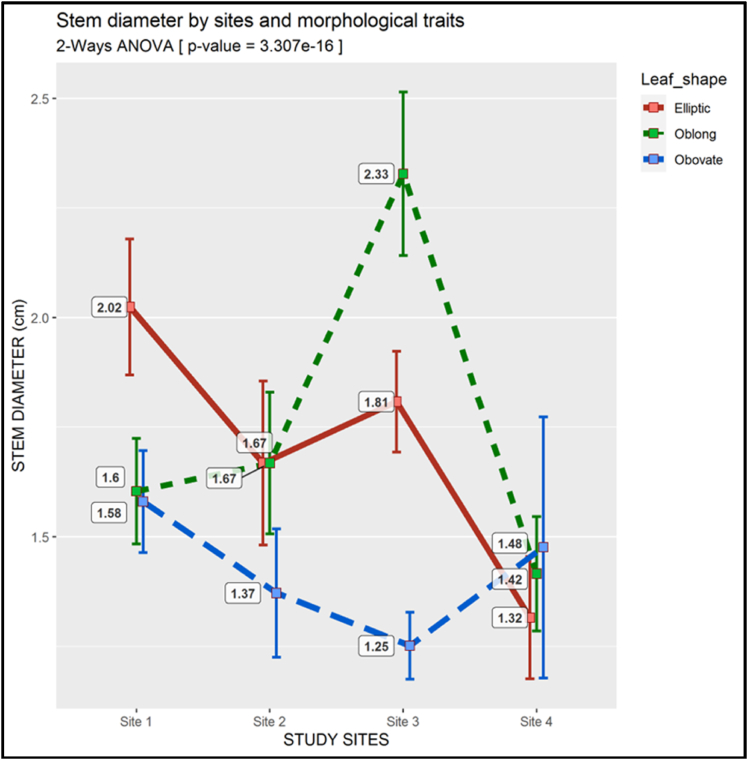
Two-way ANOVA of the Stem Diameter (There is a descriptive legend on the figure)

There was no discernible trend in stem diameter (Fig. 9). The Elliptic morphotype had the largest stem diameter (2.02 cm) at site 1. Both the Elliptic and Oblong morphotypes had the same mean stem diameter at site 2 (1.67 cm), which was also higher than that of the Obovate morphotype. The Oblong had significantly the largest mean stem diameter (ca. 2.33 cm) at site 3 whereas the Obovate dominated with an average stem diameter of 1.48 cm at site 4 (p-value <0.01).

**Fig. 10:**
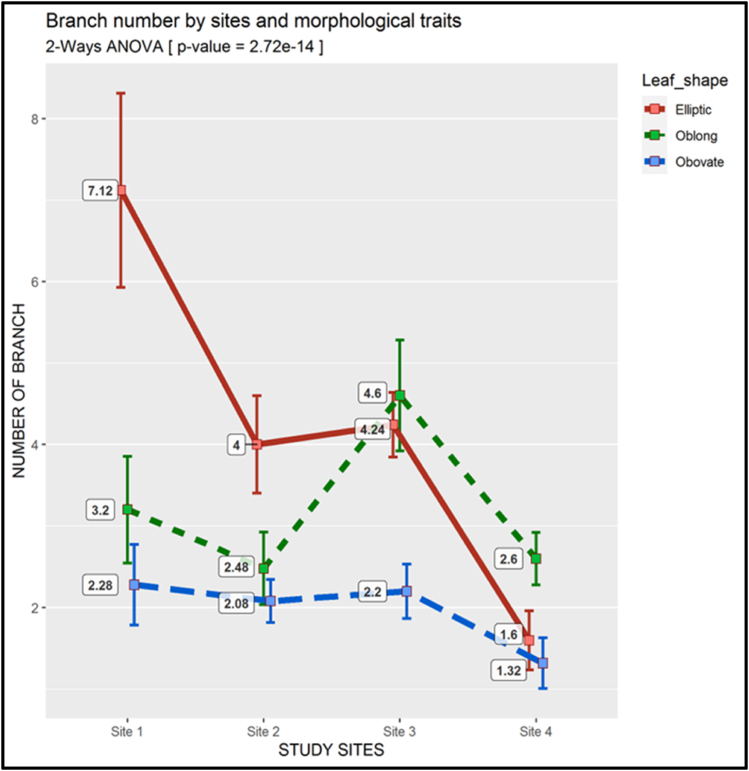
Two-way ANOVA of the Branch Number (There is a descriptive legend on the figure)

The Obovate morphotype had a significant (p*-*value <0.01) and consistently the lowest number of branches at all the study sites. At sites 1 and 2, the greatest average number of branches was 7 (7.12) with Elliptic morphotype, whereas at sites 3 and 4, the greatest average number of branches were 5 (4.6) and 3 (2.6), respectively; recorded on Oblong morphotype (Fig. 10).

**Fig. 11:**
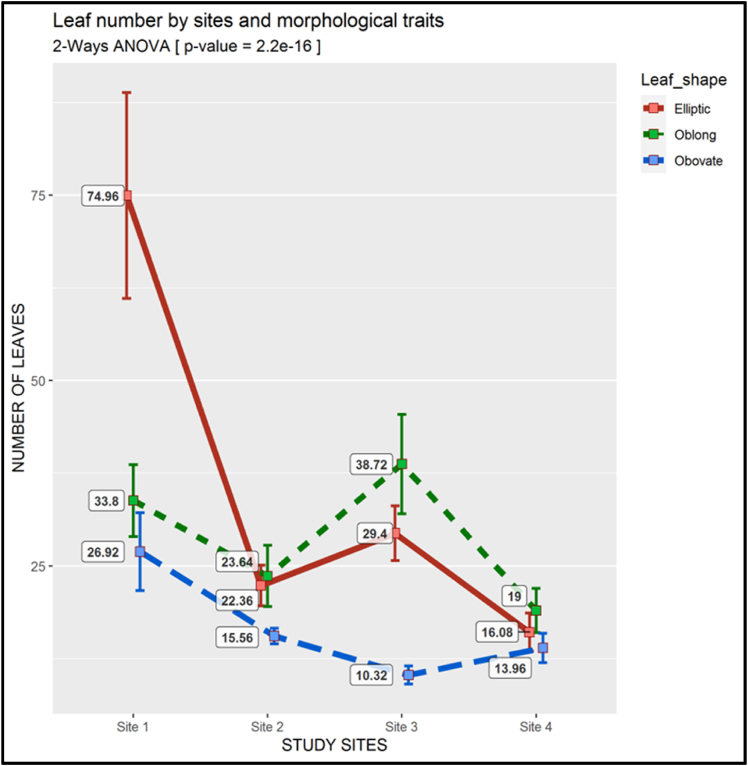
Two-way ANOVA of the Leaf Number (There is a descriptive legend on the figure)

Concerning the other quantitative features, the foliage varied greatly with the study site when compared to the morphotypes. The average number of leaves per stem for the Elliptic morphotype was 75 (74.96) at site 1. This number was markedly higher than the average number of leaves recorded in other morphotypes and remained the greatest number of leaves reported throughout the study, which decreased to 22, 29, and 16 at sites 2, 3, and 4, respectively. The Oblong morphotype dominated at sites 2, 3, and 4, with 24, 39, and 19 average leaves per stem. The Obovate morphotype consistently had the lowest number of leaves among the sites, the value of which ranged from ca. 10(10.32) at site 3 to ca. 27 (26.92) at site 1. The p*-*value for the number of leaves (2.2 *ˣ* 10^−16^) was far smaller than 0.01 (Fig. 11).

**Fig. 12:**
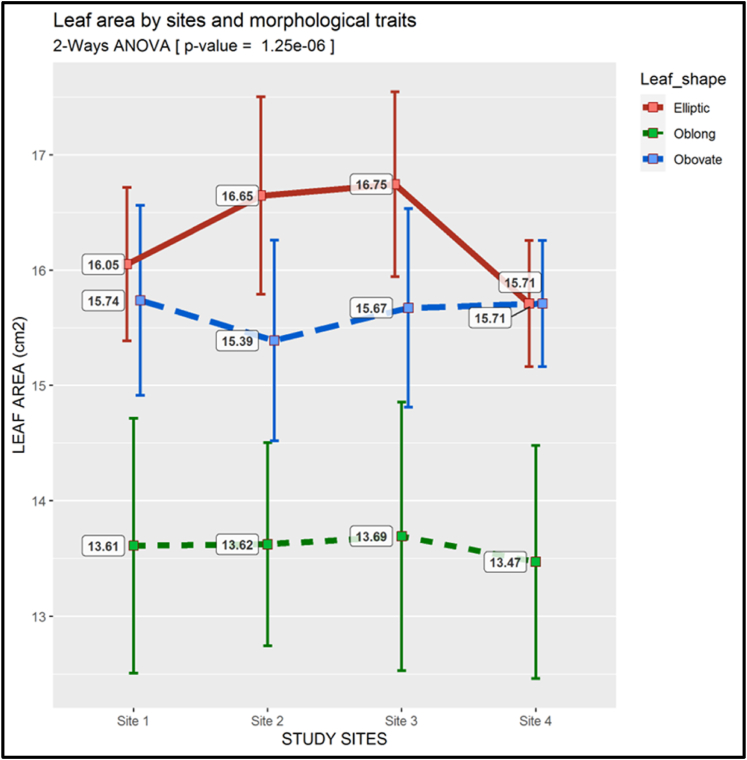
Two-way ANOVA of the Leaf area (There is a descriptive legend on the figure)

There were marked differences in leaf area between the morphotypes, in different locations. The Elliptic morphotype had the largest leaf area at sites 1, 2 and 3, but had the same average leaf area as the Obovate morphotype at site 4. The Elliptic morphotype had the biggest leaf area covering within a narrow range from ca. 16–17 cm^2^, among the four sites. The Obovate morphotype had the second biggest leaves. Its leaf area ranged from 13 to 15 cm^2^. The leaf area's p-value (1.25 ˣ 10^−6^) was smaller than 0.01 (Fig. 12).

**Fig. 13:**
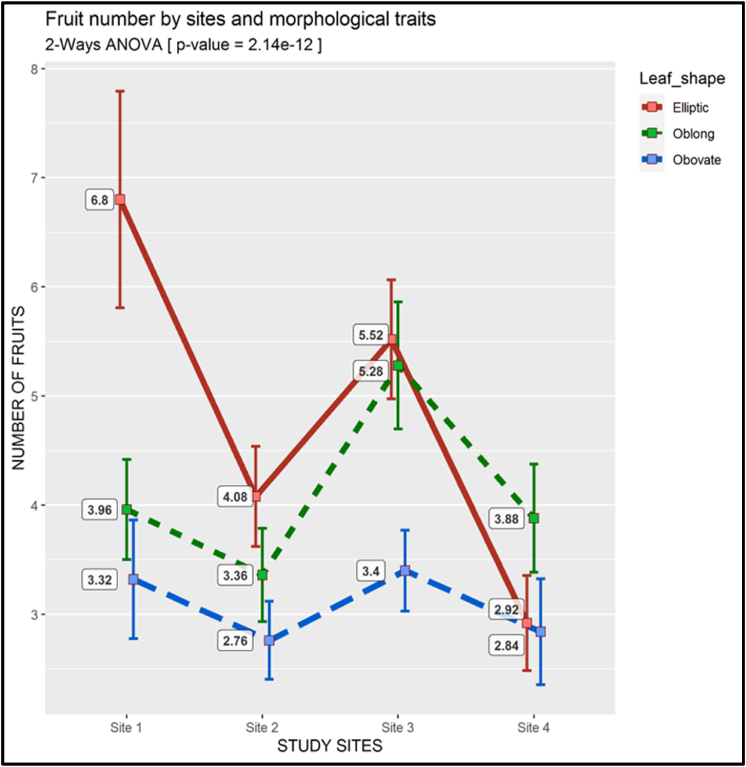
Two-way ANOVA of the Fruit number (There is a descriptive legend on the figure)

The greatest average fruit produced per plant at sites 1,2 and 3 were recorded in the Elliptic morphotype. The maximum average number of fruits for this morphotype was 7 (6.8) at site 1, which was approximately double that of the other morphotypes, which on average ranged from 3 to 4 fruits per stem. In contrast, at site 4, the Oblong morphotype had the greatest number of fruits equal to 4 (3.88). According to the results of the two-way ANOVA, the p-value *(*2.14 ˣ 10^−12^) was significantly smaller than 0.01 (Fig. 13).

### 3.6 Correlation analysis between plant quantitative features

**Fig. 14:**
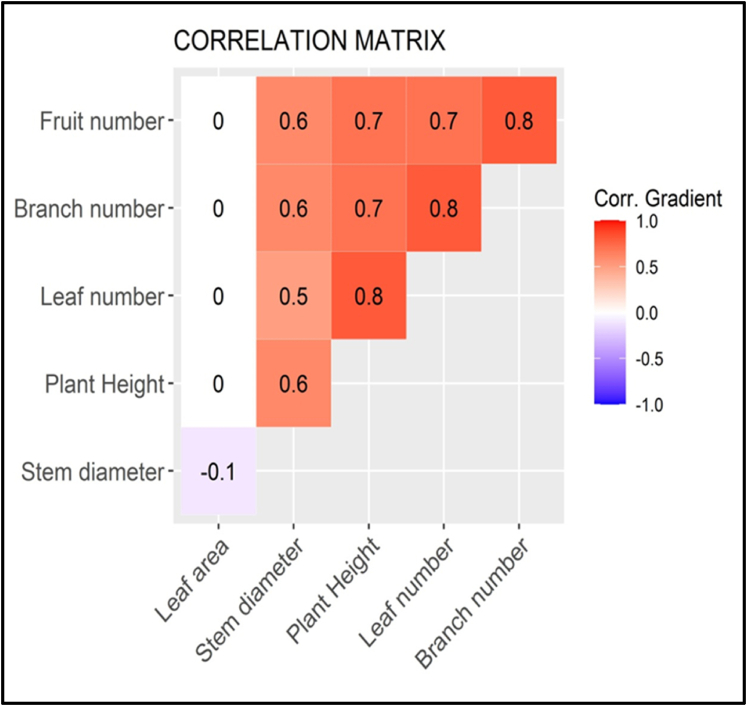
**Correlation matrix of quantitative parameters of S. kraussii**: Correlation Gradient (Corr).

A correlation analysis was performed to visualize the relationship between all the quantitative plant features (Fig. 14). The highest coefficient of correlation (CC) of 0.8, was recorded between stem height and leaf number, branch number and leaf number as well as between branch number and fruit number per upright stem. The second highest CC was 0.7, which was obtained between the stem height and branch number, stem height and fruit number, and leaf and fruit number. There was also a relatively high CC of 0.6 between the stem diameter with all parameters except with the number of leaves, with which it had a CC of 0.5. A CC of 0 was obtained between the leaf area and all traits studied, except the stem diameter with which It had a negative CC (-0.1).

## 4 Discussion

This study assessed the morphological diversity and fruit production within *Salacia kraussii* species at four sites, in the northern coast region of KwaZulu-Natal. In the current study, the leaf shape was the only qualitative morphological feature that differed markedly among *S. kraussii* individuals. Leaf morphology differences between individuals within and between populations are a common phenomenon in many plant species. It is used as an essential trait of species, varieties (cultivars), and morphotype differentiation or classification; and even evaluation of their agricultural potential [33,[47], [48], [49], [50], [51], [52], [53], [54], [55], [56]]. Thus, *S. kraussii* morphotypes were characterized by one of the three leaf shapes, named *Salacia kraussii ‘elliptic’* (Elliptic), *Salacia kraussii ‘oblong’* (Oblong) and *Salacia kraussii ‘obovate’* (Obovate). The proportions of the morphotypes varied per site, but the dominant morphotype was the Obovate and the least dominant was the Oblong. However, to make an informed decision in using the morphotypes in plant development programs, it will be prudent to investigate also the underlying genetic influence on the leaf shape.

*Salacia kraussii* plants in the northern coastal region of KZN is a suffrutex, which presented a stunted stem appearance with plant stem height averaging 30.16 cm compared to a height range of 1 m–3 m previously published (https://www.zimbabweflora.co.zw/speciesdata/species.php?species_id=137220). The study sites are sandy, poor in nutrients and characterized by acidic pH (Table 2) which could be the reasons for the short stature of the plants at the sites. In addition, the sites are used for livestock grazing. There was also evidence of fire, which is used by cattle headers to stimulate fresh grass growth. These activities can negatively impact the growth of *S. kraussii*. There is however potential for *S. kraussii* plants to grow taller as a few plants exceeding 1.5 m in height were recorded.

Comparison of plant quantitative features at various sites using one-way ANOVA, indicated *p-*values *<* 0.05 for plant height, stem diameter, number of branches, number of leaves, leaf area and the number of fruits among sites (Fig. 6) and morphotypes (Fig. 7). Results in which the *p-*values *<* 0.05 are considered statistically significant, indicating that there is strong evidence that the variation in quantitative features is a result of changes in the variables under consideration [57]. Therefore, the above-mentioned variation in quantitative features among S. *kraussii* populations is influenced by their location. The Sikhalasenkosi site (site1) was superior in terms of plant height, number of branches, number of leaves, and number of fruits. The plants performed better at Sikhalasenkosi than at the other sites because of probably two reasons. First, the Sikhalasenkosi site is in the King Cetshwayo district, located in temperate zone, which receives up to 1200 mm annually whereas KwaNdongeni (site 2), Enhlambeni (site 3) and KwaMbila (site 4) are situated in a semi-arid zone in uMkhanyakude district whose annual precipitation does not exceed 500 mm (Table 1). Second, the soil at Sikhalasenkosi was more fertile than at the other three sites (Table 2). Hence, the plants at Sikhalasenkosi are favored both in terms of rainfall and soil fertility compared to those at KwaNdongeni, Enhlambeni and KwaMbila. This could be a reflection that *S. kraaussii* is sensitive or responsive to water and fertilizer availability.

The results from the two-way ANOVA revealed significant differences among quantitative features of different morphotypes, with p-values < 0.01. This indicated that plant height, stem diameter, branch number, leaf number, leaf area and fruits number differ among plants with diverse leaf shapes. The above results show that there is significant diversity in leaf shape within the *S. kraussii* species. The Elliptic morphotype appears to be the most vigorous and productive among the four morphotypes with the greatest average values for plant height (Fig. 8), branch number (Fig. 10), foliage (Fig. 11, Fig. 12), as well as fruit number (Fig. 13). This was followed by the Oblong morphotype and lastly the Obovate morphotype. The result showed that the Elliptic morphotype dominated in terms of vegetative vigor and fruit number at site 1, located in the temperate zone. The dominance decreased the more we go to the semi-arid zone (Sites 2,3 and 4). The opposite was true for the Oblong morphotype, showing dominance in the semi-arid areas and decreasing towards the temperate zone. It seems the Elliptic morphotype performs better in areas endowed with water and the Oblong survives relatively well in the more semi-arid zone. Although the Obovate was the least in vegetative growth and fruit production, it appears to be the most dominant morphotype, with the higher plant number per plot. This reveals its ability to adapt either in temperate or arid conditions. It is suggested that information on ripe fruit characterization, including details on fruit component features and fruit biomass be gathered to also establish the diversity between morphotypes at the fruit level. There is a high probability that the three morphotypes are valid variants from which crop varieties can be developed. Genetic studies could provide valuable information regarding the diversity and the relationship between the 3 morphotypes at the molecular level.

Based on the simple correlation analysis (Fig. 14), this study also analyzed the relationship between quantitative features. The analysis indicated the existence of positive and negative relationships among quantitative features. It is known that the correlation coefficient (CC) value varies between −1 and 1, a value of ±1 shows a perfect link between the researched features [[54], [55], [56]]. The results revealed that the upright stem height was closely related to leaf number. The same was observed for the branch number-leaf number and branch number-fruits number per *S. kraussii* stem (CC = 0.8). The correlation between other parameter pairs such as the height of plants-number of branches, the height of plants-number of fruits and the number of leaves-number of fruits was also relatively high (CC = 0.7). These results mirrored the high correlation between plant height-fruits biomass and stem diameter-fruits biomass observed for *Strychnos madagascariensis* and *Strychnos spinosa* growing in the same study area, even though the analysis was based on fruits biomass instead of fruit number [57]. Similarly, as was recently observed with *Trichilia emetica* from the same location, the stem diameter was substantially positively linked with fruit number [58]. A high positive average correlation was also identified between stem diameter and all other features measured (CC = 0.6), except for the number of leaves, where it was a bit lower (CC = 0.5). Unlike most quantitative features, there was no positive correlation between the leaf area and other variables (CC = 0). Surprisingly, there was a negative correlation between the leaf area and the stem diameter (CC = − 0.1).

This study has potential limitations. Given that the survey data were collected on wild population located in open areas exposes the study plants to multiple contact with the local human population (and animals) that might harvest some of the fruits before our arrival resulting in fewer fruits recorded per plant than was produced by the plants. Therefore, data on fruit production of *S. kraussii* morphotypes reported here may not reflect the true fruit production potential of the plants. For further studies assessing fruit production in controlled areas or under cultivated conditions would provide a better evaluation of fruit production by S. *kraussii*.

Although this was not part of the study, the major threats identified are the degradation of *S. kraussii natural* habitats due to intensive grazing by cattle and goats (and zebra), as well as the use of fire by cattle herders to manage the pastures. In addition to eating the foliage, the goats and cattle would browse the plants and eat the ripe fruits, which may negatively impact the growth, fruit productivity, and seedling recruitment. The protection of *S. kraussii* habitats and its domestication would be the most effective conservation and extinction prevention measures.

## 5 Conclusion

This research revealed the existence of three morphotypes within the wild species *S. kraussii* growing on the northern coast of Kwazulu-Natal. Morphotype with obovate leaves, designated *‘S. kraussii obovate*’, is well represented in all study sites. This reveals its high tolerance to temperate and semi-arid conditions. However, it has fewer fruit production than others. Morphotype with elliptic leaves, *‘S. kraussii elliptic*’, is the most vigorous and productive form, which dominates the temperate site with fewer representatives in the semi-arid sites. In contrast morphotype with oblong leaves, *‘S. kraussii oblong*’ is more dominant in the semi-arid sites.

## Funding

This research was supported by the BEBUC Scholarship Program and the Else Kröner-Fresenius-Stiftung (And partners): www.foerderverein-uni-kinshasa.de. The authors gratefully acknowledge BEBUC's moral and scientific support.

## Data availability

The data (raw data) of this article can be obtained upon request.

## Additional information

No additional information is available pertaining to this article.

## CRediT authorship contribution statement

**Merveille Mukaya Kisepa:** Writing – original draft, Methodology, Investigation, Formal analysis, Conceptualization. **Elijah Godfrey Zharare:** Writing – original draft, Validation, Supervision, Investigation, Conceptualization. **Clemence Zimudzi:** Validation, Conceptualization. **Arindo Lukawu Akweni:** Methodology, Formal analysis.

## Declaration of competing interest

The authors declare that they have no known competing financial interests or personal relationships that could have appeared to influence the work reported in this paper.

## References

[1] W T., V. B J. (2013). Climate change impacts on global food security. Science.

[2] Frison E.A., Cherfas J., Hodgkin T. (2011). Agricultural biodiversity is essential for a sustainable improvement in food and nutrition security. Sustainability.

[3] Leridon H. (2020). World population outlook: EXPLOSION OR IMPLOSION? Electronic distribution by Cairn on behalf of I. Populations & Societies.

[4] Bharucha Z., Pretty J. (2010). The roles and values of wild foods in agricultural systems. Phil. Trans. Biol. Sci..

[5] Fong B.Y.F., Chiu W.K., Chan W.F.M., Lam T.Y. (2021). A review study of a green diet and healthy ageing. Int. J. Environ. Res. Publ. Health.

[6] Mazza E. (2021). Mediterranean diet in healthy aging. J. Nutr. Health Aging.

[7] Yeung S.S.Y., Kwan M., Woo J. (2021). Healthy diet for healthy aging. Nutrients.

[8] Hickey G.M., Pouliot M., Smith-Hall C., Wunder S., Nielsen M.R. (2016). Quantifying the economic contribution of wild food harvests to rural livelihoods: a global-comparative analysis. Food Pol..

[9] Govender L., Pillay K., Siwela M., Modi A., Mabhaudhi T. (2017). Ssfood and nutrition insecurity in selected rural communities of KwaZulu-Natal, South Africa—linking human nutrition and agriculture. Int. J. Environ. Res. Publ. Health.

[10] Bvenura C., Sivakumar D. (2017). The role of wild fruits and vegetables in delivering a balanced and healthy diet. Food Res. Int..

[11] Khumalo G.P., Van Wyk B.E., Feng Y., Cock I.E. (2022). A review of the traditional use of southern African medicinal plants for the treatment of inflammation and inflammatory pain. J. Ethnopharmacol..

[12] Van Wyk B.-E. (2017).

[13] Van Wyk B.E. (2015). A review of commercially important African medicinal plants. J. Ethnopharmacol..

[14] Nkosi N.N., Mostert T.H.C., Dzikiti S., Ntuli N.R. (2020). Prioritization of indigenous fruit tree species with domestication and commercialization potential in KwaZulu-Natal, South Africa. Genet. Resour. Crop Evol..

[15] Omotayo A.O., Aremu A.O. (2020). Underutilized African indigenous fruit trees and food–nutrition security: opportunities, challenges, and prospects. Food Energy Secur..

[16] Chawafambira A., Sedibe M.M., Mpofu A., Achilonu M. (2020). Uapaca kirkiana, an indigenous fruit tree in sub-Saharan Africa: a comprehensive review. Cogent Food Agric..

[17] Van Wyk B.E. (2015). A review of commercially important African medicinal plants. J. Ethnopharmacol..

[18] Magaia T., Uamusse A., Sjöholm I., Skog K. (2013). Proximate analysis of five wild fruits of Mozambique. Sci. World J..

[19] Zimila H.E. (2020). Phytochemical analysis and in vitro antioxidant and antimicrobial activities of hydroalcoholic extracts of the leaves of Salacia kraussii. Biocatal. Agric. Biotechnol..

[20] Figueiredo J.N., Räz B., Séquin U. (1998). Novel quinone methides from Salacia kraussii with in vitro antimalarial activity. J. Nat. Prod..

[21] Karagöz A., Pilanali N., Polat T. (2006). Agro-morphological characterization of some wild wheat (Aegilops L. and Triticum L.) species. Turk. J. Agric. For..

[22] Toklu F., Tuba Biçer B., Karaköy T. (2009). Agro-morphological characterization of the Turkish lentil landraces. Afr. J. Biotechnol..

[23] Nooryazdan H., Serieys H., Baciliéri R., David J., Bervillé A. (2010). Structure of wild annual sunflower (Helianthus annuus L.) accessions based on agro-morphological traits. Genet. Resour. Crop Evol..

[24] Belaj A., León L., Satovic Z., De la Rosa R. (2011). Variability of wild olives (Olea europaea subsp. europaea var. sylvestris) analyzed by agro-morphological traits and SSR markers. Sci. Hortic..

[25] del Carmen Morales Saavedra J., Rodríguez Zaragoza F.A., Cabrera Toledo D., Sánchez Hernández C.V., Vargas-Ponce O. (2019). Agromorphological characterization of wild and weedy populations of Physalis angulata in Mexico. Sci. Hortic..

[26] Harouna D.V., Venkataramana P.B., Matemu A.O., Alois Ndakidemi P. (2020). Agro-morphological exploration of some unexplored wild vigna legumes for domestication. Agronomy.

[27] Ilhan G., Gundogdu M., Karlović K., Židovec V., Vokurka A., Ercişli S. (2021). Main agro-morphological and biochemical berry characteristics of wild-grown sea buckthorn (Hippophae rhamnoides L. ssp. caucasica rousi) genotypes in Turkey. Sustainability.

[28] Subaşı I. (2022). Agro-morphological characterization and some seed characteristics of wild crambe (Brassicaceae) species in Turkey. Sustainability.

[29] Chakraborty S., Mukherjee D., Baskey S. (2015). Paradigm of demographic stochasticity-way to extinction of valeriana jatamansi jones, a valuable medicinal plant in north eastern himalayan region. Ecol. Environ. Conserv..

[30] Cheng F., Wu J., Liang J., Wang X. (2015). The Brassica Rapa Genome.

[31] Hayano-Kanashiro C., Gámez-Meza N., Medina-Juárez L.Á. (2016). Wild Pepper Capsicum annuum L. var. glabriusculum: taxonomy, plant morphology, distribution, genetic diversity, genome sequencing, and phytochemical compounds. Crop Sci..

[32] Razo-Mendivili F.G., Hernandez-Godínez F., Kanashiro C.H., Martínez O. (August, 2021). Transcriptomic analysis of a wild and acultivated varieties of Capsicum annuum over fruit development and ripening. PLoS One.

[33] Hoper J.M.I., Gourlay C.W., Noel Ellis T.H. (2001). Genetic control of leaf morphology: a partial view. Ann. Bot..

[34] Sheidai M., Ziaee S., Farahani F., Talebi S.M., Noormohammadi Z., Farahani Y.H.A. (2014). Infra-specific genetic and morphological diversity in Linum album (Linaceae). Biologia (Poland).

[35] Dorice L.L., Ephraim J.M., George M.M. (2020). A review of plant characterization: first step towards sustainable forage production in challenging environments. Afr. J. Plant Sci..

[36] Botha G., Porat N. (2007). Soil chronosequence development in dunes on the southeast African coastal plain, Maputaland, South Africa. Quat. Int..

[37] Mucina L. (2018).

[38] Peel M.C., Finlayson B.L., McMahon T.A. (2007). Updated world map of the Köppen-Geiger climate classification. Hydrol. Earth Syst. Sci..

[39] Engelbrecht C.J., Engelbrecht F.A. (2016). Shifts in Köppen-Geiger climate zones over southern Africa in relation to key global temperature goals. Theor. Appl. Climatol..

[40] Beck H.E., Zimmermann N.E., Mcvicar T.R., Vergopolan N., Berg A., Wood E.F. (2018).

[41] Beck H.E., Zimmermann N.E., McVicar T.R., Vergopolan N., Berg A., Wood E.F. (2020). Publisher Correction: present and future Köppen-Geiger climate classification maps at 1-km resolution. Sci. Data.

[42] Grove A.T., Miles M.R., Worthington E.B., Doggett H., Dasgupta B., Farmer B.H. (1977). The geography of semi-arid lands [and discussion]. Phil. Trans. Biol. Sci..

[43] Ellis B. (2009).

[44] Christenhusz M.J.M. (2010). The Kew Plant Glossary, an illustrated dictionary of plant terms. Bot. J. Linn. Soc..

[45] (2011). The Kew plant glossary: an illustrated dictionary of plant terms. Choice Reviews Online.

[46] Snow N. (2011). The kew plant glossary: an illustrated dictionary of plant terms. Syst. Bot..

[47] Asare P.A., Galyuon I.K.A., Sarfo J.K., Tetteh J.P. (2011). Morphological and molecular based diversity studies of some cassava (Manihot esculenta crantz) germplasm in Ghana. Afr. J. Biotechnol..

[48] Wang H., Wang R., Harrison S.P., Prentice I.C. (2022). Leaf morphological traits as adaptations to multiple climate gradients. J. Ecol..

[49] Yang J. (2015). Leaf form-climate relationships on the global stage: an ensemble of characters. Global Ecol. Biogeogr..

[50] Zhu J. (Jan. 2022). Variation in leaf morphological traits of European beech and Norway spruce over two decades in Switzerland. Frontiers in Forests and Global Change.

[51] Bhatia N., Runions A., Tsiantis M. (2021). Leaf shape diversity: from genetic modules to computational models. Annu. Rev. Plant Biol..

[52] Rowland S.D. (2020). Leaf shape is a predictor of fruit quality and cultivar performance in tomato. New Phytol..

[53] Dornbusch T., Watt J., Baccar R., Fournier C., Andrieu B. (2011). A comparative analysis of leaf shape of wheat, barley and maize using an empirical shape model. Ann. Bot..

[54] Mbhele Z., Zharare G.E., Zimudzi C., Ntuli N.R. (Jun. 2022). Indigenous knowledge on the uses and morphological variation among Strychnos spinosa lam. At oyemeni area, KwaZulu-natal, South Africa. Sustainability.

[55] Mbhele Z., Zharare G.E., Zimudzi C., Ntuli N.R. (2022). Morphological variation of Strychnos spinosa lam. Morphotypes: a case study at bonamanzi game reserve, KwaZulu-natal, South Africa. Diversity.

[56] Chattopadhyay S., Tikader A., Das N.K. (2011). Nondestructive, simple, and accurate model for estimation of the individual leaf area of som (Persea bombycina). Photosynthetica.

[57] Held L., Ott M. (2018). On p-values and bayes factors. Annu Rev Stat Appl.

